# Molecular Adjuvant Ag85A Enhances Protection against Influenza A Virus in Mice Following DNA Vaccination

**DOI:** 10.3390/v4123606

**Published:** 2012-12-10

**Authors:** Jun Dai, Decui Pei, Baoning Wang, Yu Kuang, Laifeng Ren, Kang Cao, Huan Wang, Bin Zuo, Jingjing Shao, Sha Li, Hong Li, Mingyuan Li

**Affiliations:** 1 Department of Microbiology, West China School of Preclinical and Forensic Medicine, Sichuan University, Chengdu, Sichuan 610041, China; 2 Department of Pathobiology and Immunology, School of Basic Medicine, Hebei Medical University, Shijiazhuang, Hebei 050091, China; 3 The Joint Research Center of West China Second University Hospital of Sichuan University and the Faculty of Medicine, University of Hong Kong, Sichuan University, Chengdu, Sichuan 610041, China; 4 State Key Laboratory of Oral Diseases, Sichuan University, Chengdu, Sichuan 610041, China

**Keywords:** DNA vaccine, influenza A virus, *Mycobacterium tuberculosis* secreted antigen Ag85A, HA2 epitope

## Abstract

A novel DNA vaccine vector encoding the *Mycobacterium tuberculosis* secreted antigen Ag85A fused with the influenza A virus (IAV) HA2 protein epitopes, pEGFP/Ag85A-sHA2 (pAg85A-sHA2), was designed to provide protection against influenza. The antigen encoded by the DNA vaccine vector was efficiently expressed in mammalian cells, as determined by reverse transcription polymerase chain reaction (RT-PCR) and fluorescence analyses. Mice were immunized with the vaccine vector by intramuscular injection before challenge with A/Puerto Rico/8/34 virus (PR8 virus). Sera and the splenocyte culture IFN-γ levels were significantly higher in immunized mice compared with the control mice. The novel vaccine group showed a high neutralization antibody titer *in vitro*. The novel vaccine vector also reduced the viral loads, increased the survival rates in mice after the PR8 virus challenge and reduced the alveolar inflammatory cell numbers. Sera IL-4 concentrations were significantly increased in mice immunized with the novel vaccine vector on Day 12 after challenge with the PR8 virus. These results demonstrated that short HA2 (sHA2) protein epitopes may provide protection against the PR8 virus and that Ag85A could strengthen the immune response to HA2 epitopes, thus, Ag85A may be developed as a new adjuvant for influenza vaccines.

## 1. Introduction

The influenza A virus (IAV) pandemic of 2009 involving the H1N1 strain has raised international concern over the potential for outbreaks of increased severity and highlighted the need for improved global surveillance and vaccination strategies [1,2]. The most effective way to prevent IAV infection is through vaccination. The proteins of IAV are used for vaccination. However, these vaccines need to be updated annually because IAVs are constantly undergoing change. It is therefore of substantial interest to develop a vaccine that targets conserved viral proteins (e.g., M2e, NP) and could be used to protect against unpredicted antigenic variation in both epidemic and pandemic outbreaks. These conserved viral protein vaccines do not usually target the surface molecules of IAV. This has resulted in a substantial drop in the efficacy of vaccination. It is important to develop a vaccine that targets the surface molecule of IAV.

Hemagglutinin (HA) is the most abundant protein on the viral coat and is highly immunogenic. Antibody responses against the viral surface protein HA are the major determinants of protection against IAV [3,4]. However, variations in HA arise rapidly due to antigenic shift and drift, allowing the virus to evade the immunity conferred by seasonal vaccines [5]. HA is synthesized as the precursor HA0, which is cleaved into the HA1 and HA2 subunits. Whereas the membrane distal domain (HA1) can be highly variable, the stalk region containing the core fusion machinery constituted primarily by the HA2 subunit is relatively conserved [6,7]. Therefore, the development of an IAV vaccine promoting enhanced immune responses to these conserved IAV domains should provide more effective protection against this rapidly evolving virus. Antibodies targeting the HA2 subunit can provide broad protection against both seasonal and pandemic IAV infections [7]. However, HA2 is masked by the membrane-distal portion of HA, a bulky and highly immunogenic globular head domain [8]. Generation of broadly neutralizing antibodies (NAbs) is difficult because the use of the conserved fusion peptide of HA alone as a vaccine is only weakly immunogenic and does not confer adequate protection against IAV [8]. Therefore, strategies to boost immune responses targeting HA2 are needed. 

The Bacille Calmette-Guérin (BCG) vaccine against *Mycobacterium tuberculosis* (*M. tuberculosis*) has demonstrated marked immunomodulatory effects and enhanced antigen-specific antibody production when used in combination with influenza vaccines [9,10,11]. As a major secreted antigen of M. tuberculosis and an immunodominant antigen of BCG, *M. tuberculosis* secreted antigen Ag85A (Ag85A) is known to increase T helper type 1 (Th1) cytokine responses [12], such as interferon (IFN)‑γ, which are important for cell-mediated immunity against IAV [13].

Based on the hypothesis that the Ag85A may serve as an effective immune adjuvant for HA2, we constructed a novel influenza vaccine vector expressing the conserved region of the HA protein linked to Ag85A. The efficacy of this vaccine vector in preventing mortality and morbidity in mice was evaluated after challenge with IAV, and the cytokine profiles of the sera and cultured splenocytes were determined.

## 2. Results

### 2.1. Expression of Ag85A-sHA2 in Eukaryotic Cells Transfected with the Plasmid DNA Vectors

**Figure 1:**
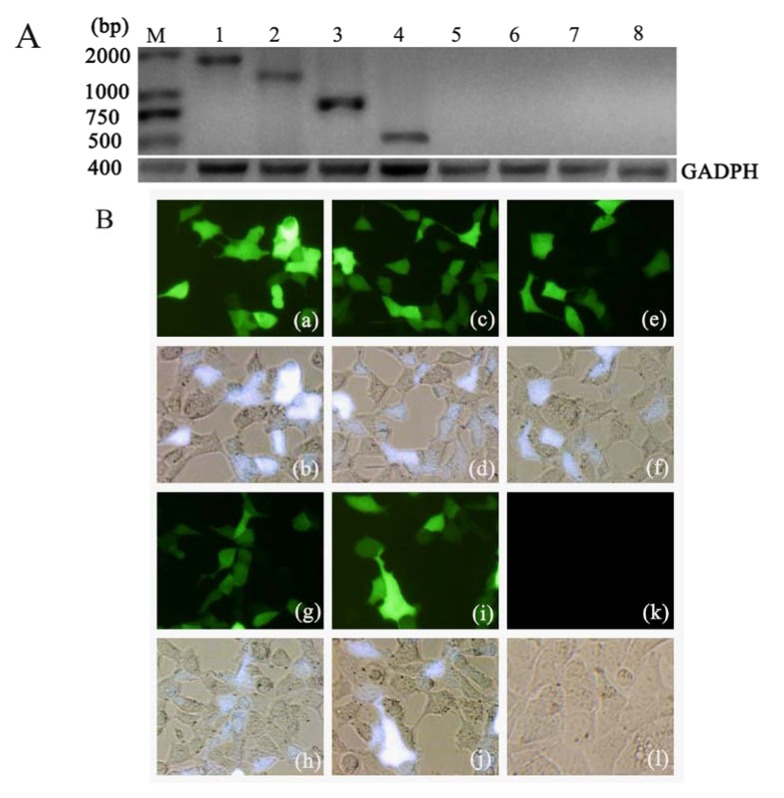
Expression of HA, sHA2, Ag85A or Ag85A-sHA2 from DNA vaccine vectors in HEK293 cells. (**A**) Expression was evaluated by reverse transcription polymerase chain reaction (RT-PCR) as follows: (1) cells transfected with pHA; (2) cells transfected with pAg85A-sHA2; (3) cells transfected with pAg85A; (4) cells transfected with psHA2; (5)–(8) cells transfected with pEGFP-C2; (M) Trans2K Plus DNA Marker. Fragments of approximately 1,700 bp (1), 1,250 bp (2), 750 bp (3) 500 bp (4) and 400 bp (1)–(8) were separated by 2% agarose gel electrophoresis following RT-PCR. GADPH: fragments of approximately 400 bp; pAg85A: fragments of approximately 750 bp; psHA2: fragments of approximately 500 bp; pAg85A-sHA: fragments of approximately 1,250 bp; pHA: fragments of approximately 1,700 bp. (**B**) Expression of HA2 or Ag85A in HEK293 cells. (400×) HEK293 cells were transfected with plasmids encoding the individual HA, sHA2 or Ag85A proteins or the Ag85A-sHA2 fusion protein, and their expression levels were evaluated by green fluorescence protein (GFP) fluorescence. The images show cells that were transfected with pHA (a) and (b), pAg85A-sHA2 (c) and (d), pAg85A (e) and (f), psHA2 (g) and (h), pEGFP-C2 (i) and (j) or mock-transfected cells (k) and (l). Fluorescent images (a), (c), (e), (g), (i) and (k) and phase contrast images (b), (d), (f), (h), (j) and (l) are shown.

The transfection of HEK293 cells with the DNA vaccine vectors, pHA, pAg85A-sHA2, pEGFP-C2, pAg85A and psHA2, all produced strong green fluorescent signals (Figure 1A). The reverse transcription polymerase chain reaction (RT-PCR) results (Figure 1B) also confirmed that pHA, pAg85A-sHA2, pAg85A and psHA2 successfully expressed the expected transcripts in the transfected HEK293 cells. These results indicated that the HA, Ag85A, sHA2 and the Ag85A-sHA2 fusion genes could be transiently expressed in HEK293 cells.

### 2.2. Vaccine-Mediated Induction of Functional Antibody Titers and Effect on IAV Challenge

Mice were immunized with pAg85A-sHA2, psHA2, pAg85A, PBS, pEGFP-C2 or pHA. Development of antibodies against HA was evaluated by hemagglutination inhibition (HI) assays. The HI titers were represented as group arithmetic means (log 2 dilution). As shown in Table 1, the highest HI titer against the PR8 virus was detected in mice immunized with pHA whose mean titers increased by approximately 1.5-fold after boosting. However, HI activity against the PR8 virus was not detected in mice immunized with psHA2 or pAg85A-sHA2. 

**Table 1:** Comparison of antibody titers (log 2 dilution) in hemagglutination inhibition assays.

Group	Primary response	Secondary response
pHA	4.00 ± 0.82	5.75 ± 0.95
pAg85A-sHA2	2.50 ± 0.58	2.75 ± 0.50
pAg85A	1.75 ± 0.96	1.75 ± 0.96
psHA2	2.25 ± 0.50	2.50 ± 0.58
pEGFP-C2	1.50 ± 0.58	1.50 ± 0.58
PBS	1.50 ± 0.58	1.50 ± 0.58

**Figure 2:**
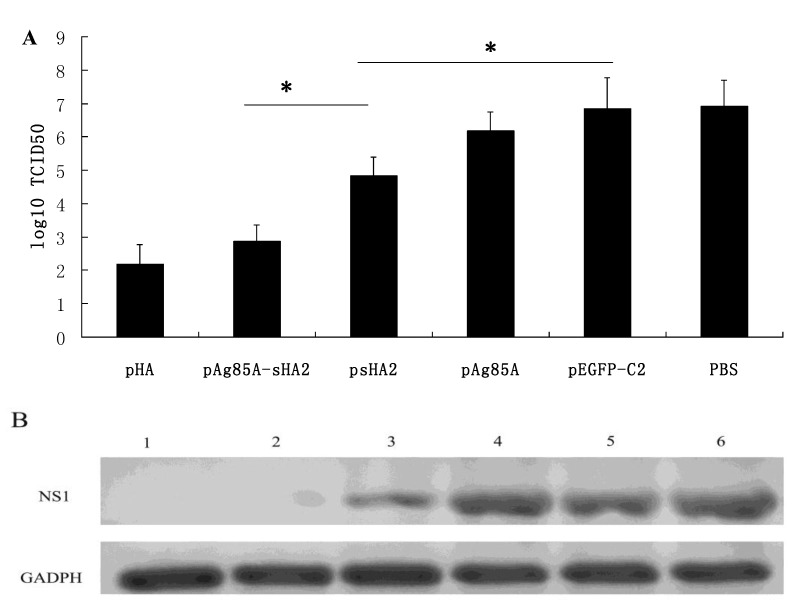
Comparison of lung viral loads on Day 8 after PR8 virus challenge. (**A**) Comparison of lung viral loads by TCID50. The IAV titers from the samples from the pEGFP/Ag85A-sHA2 vaccinated group were lower than those from groups vaccinated with the plasmid vaccines pEGFP-C2, psHA2 or pAg85A. Mice that were immunized with pHA, psHA2 or pAg85A-sHA2 all showed markedly decreased lung PR8 viral loads compared with the pAg85A, PBS and pEGFP-C2 control groups (* *P *< 0.05). (**B**) Comparison of lung viral load by RT-PCR. 1, pHA; 2, pAg85A-sHA2; 3, psHA2; 4, pEGFP-C2; 5, pAg85A; 6, PBS.

However, the immunization of mice with pHA, psHA2 or pAg85A-sHA2, all showed an significant decrease in lung PR8 virus loads in mice compared with the PBS and pEGFP-C2 immunized control groups (Figure 2) (*P *< 0.05). Furthermore, the IAV titers in the samples from the pAg85A-sHA2 vaccinated group were significantly lower than those in the other groups vaccinated with the plasmid vaccine vectors pEGFP-C2, psHA2 and pAg85A (*P *< 0.05). While not as effective as pAg85A-sHA2, vaccination with psHA2 decreased the challenge viral load to a significantly lower level compared with the pEGFP-C2 and pAg85A vaccinated groups (*P *< 0.05). 

**Figure 3:**
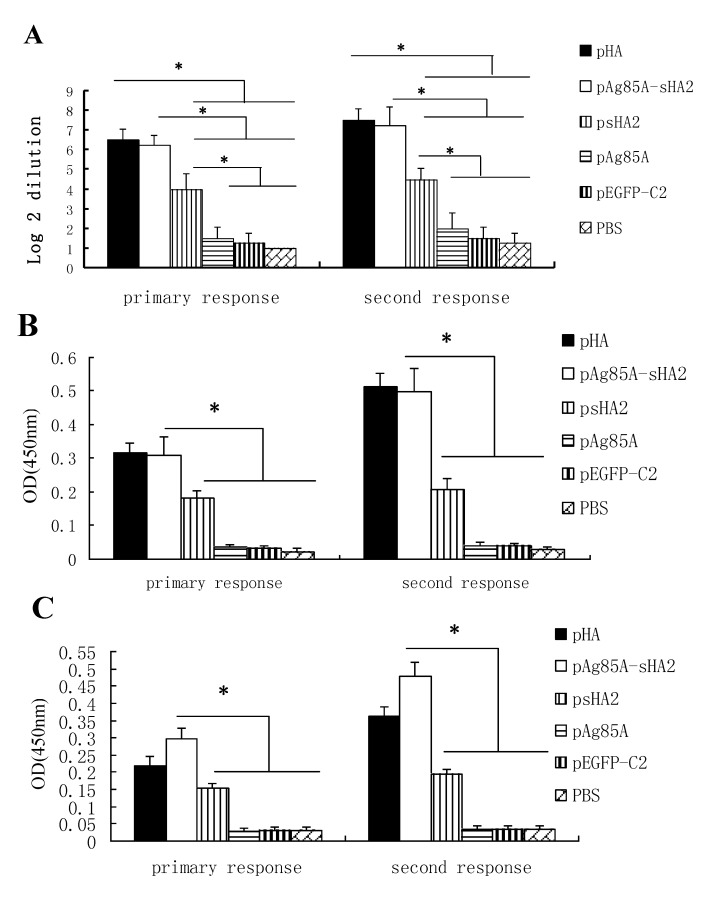
Comparison of serum antibody titers (* *P *< 0.05) (**A**) Comparison of serum antibody titers (log 2 dilution) conferring 50% cytopathic effect (CPE) inhibition as assayed in Madin-Darby canine kidney (MDCK) cells. High neutralizing antibodies (Nab) titers were detected in mice immunized with pHA, pAg85A-sHA2 or psHA2. The NAb titer from the pAg85A-sHA2 vaccinated group was higher than those from the PBS, pEGFP-C2, psHA2 and pAg85A vaccinated groups (*P *< 0.05). The NAb titer from the pHA vaccinated group was higher than those from the PBS, pEGFP-C2, psHA2 and pAg85A vaccinated groups (*P *< 0.05). The NAb titer from the psHA2 vaccinated group was higher than those from the PBS, pEGFP-C2 and pAg85A vaccinated groups (*P *< 0.05). (**B**) Comparison of serum antibody titers by ELISA of recombinant H1 HA proteins. The ELISA substrates used were indicated for recombinant HA proteins of A/PR/8/34. (**C**) Comparison of serum antibody titers by ELISA of recombinant H5 HA proteins. The ELISA substrates used were indicated for recombinant H5 HA proteins of A/Anhui/1/2005.

The development of NAbs against HA was evaluated by the neutralization assay. The titers from each serum sample were expressed as the dilutions (log 2 transformed) that reduced the cytopathic effect (CPE) resulting from the viral inoculation by 50% in the Madin-Darby canine kidney (MDCK) cells. As shown in Figure 3A, high NAb titers were detected in mice immunized with pHA, pAg85A-sHA2 and psHA2. The NAb titer from the pAg85A-sHA2 vaccinated group was higher than those from the pEGFP-C2, psHA2 and pAg85A vaccinated groups (*P *< 0.05). The NAb titer from the psHA2 vaccinated group was significantly higher than those from the pEGFP-C2, PBS and pAg85A vaccinated groups (*P* < 0.05).

The titers of IgG against HA were evaluated by ELISA. The titers of each serum sample were expressed as optical density (OD) values. The ELISA substrates used were indicated for recombinant H1 HA proteins of A/PR/8/34 (Figure 3B) or recombinant H5 HA proteins of A/Anhui/1/2005 (Figure 3C). As shown in Figure 3B,C, the antibody titer from the pAg85A-sHA2 vaccinated group was significantly higher than those from the pEGFP-C2, psHA2 and pAg85A vaccinated groups (*P *< 0.05). The IgG NAb titer from the pAg85A-sHA2 vaccinated group was significantly higher than that from the psHA2 vaccinated group (Figure 3C) (*P *< 0.05).

### 2.3. Cytokine Measurements

**Figure 4:**
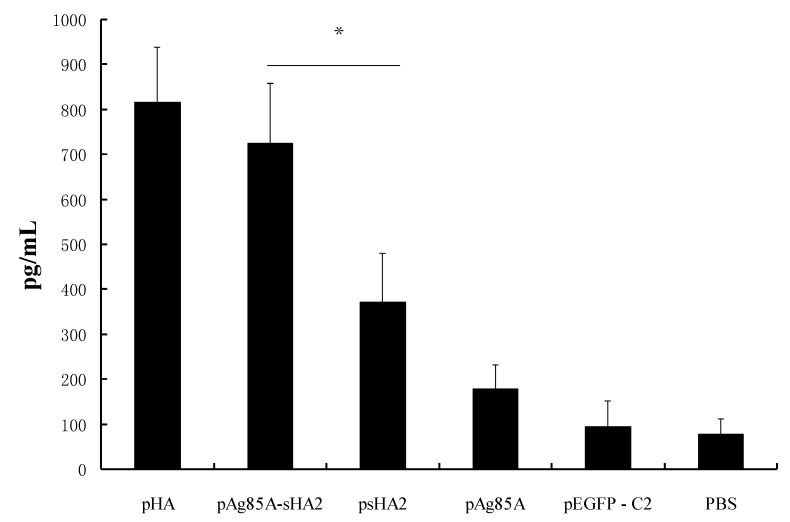
IFN-γ production in splenocyte culture at 72 h following induction by HA *in vitro*. (* *P *< 0.05) Splenocytes from mice vaccinated with pAg85A-sHA2 produced a 1.5-fold significantly higher IFN-γ response than those from mice vaccinated with psHA2.

The HA-induced IFN-γ production *in vitro* was evaluated on Day 8 after the virus challenge. IFN-γ was induced by HA or ConA *in vitro*. HA was found to induce significantly higher IFN-γ levels in the presence of pAg85A-sHA2 or pHA (Figure 4) (*P *< 0.05). Splenocytes from mice vaccinated with pAg85A-sHA2 produced a 1.5-fold significantly higher IFN-γ response to HA than those from mice vaccinated with psHA2 (*P *< 0.05). Meanwhile, IFN-γ production from the splenocytes in the PBS group was significantly lower than that of other groups upon stimulation with HA (*P *< 0.05). HA induced significantly lower IFN-γ levels than did ConA (data not shown). 

**Figure 5:**
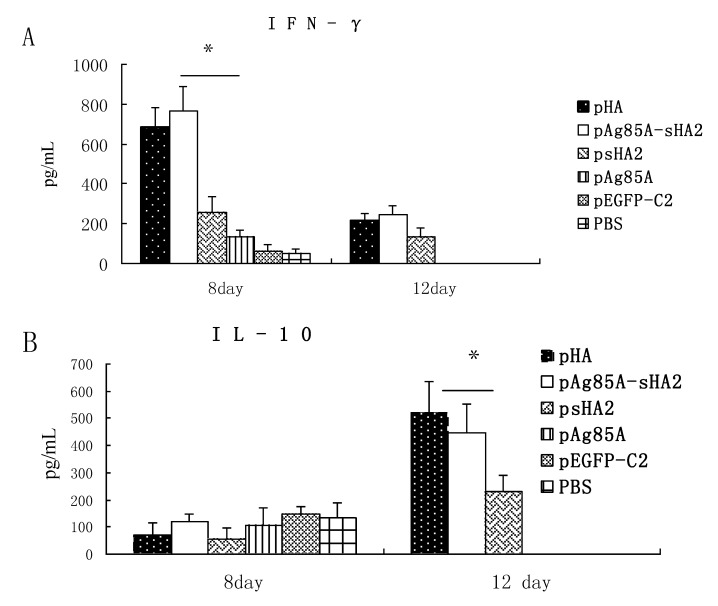
Cytokine levels in sera. (**A**) IFN-γ on Days 8 and 12 after infection with PR8 virus (* *P* < 0.05). Sera IFN-γ levels were significantly higher in the pAg85A-sHA2 vaccinated group compared with all other groups on day 8 after infection with PR8 virus, whereas the levels in the psHA2 group were significantly higher than those in the pAg85A, pEGFP-C2 and PBS groups. Data are not shown for n < 4 mice. (**B**) IL-4 on Days 8 and 12 after infection with PR8 virus (* *P* < 0.05). Sera IL-4 levels were significantly higher in the pAg85A-sHA2 group and the pHA vaccinated group than in the psHA2 group on Day 12 after infection with the PR8 virus. Data are not shown for n < 4 mice.

IL-4 and IFN-γ were measured in the sera on Days 8 and 12 after infection with the PR8 virus. Sera IFN-γ levels were significantly higher in the pAg85A-sHA2 vaccinated group compared with all other groups on Day 8 after infection with the PR8 virus, whereas the levels in the psHA2 group were significantly higher than those in the pAg85A, pEGFP-C2 and PBS groups (Figure 5A). However, no significant differences were observed in the sera IL-4 concentrations among all groups on Day 8 after challenge with IAV (*P *> 0.05) (Figure 5B). Sera IL-4 levels were significantly higher in the pAg85A‑sHA2 group or the pHA vaccinated group than those in the psHA2 group on Day 12 after infection with the PR8 virus (*P* < 0.05) (Figure 5B).

### 2.4. Comparison of Survival Rates and Histopathological Analyses

**Figure 6:**
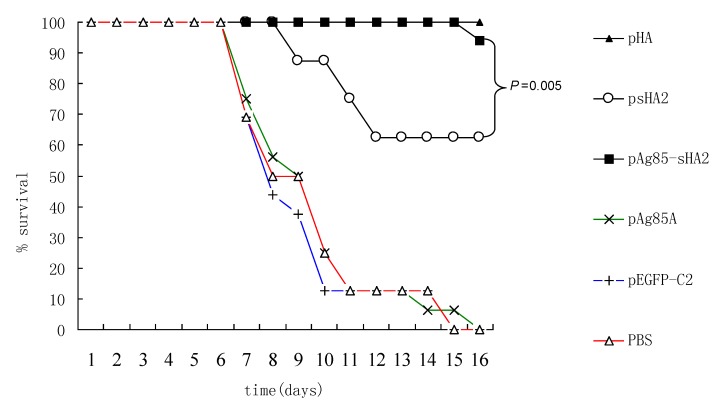
Survival rates of mice. The survival rates of mice vaccinated with pAg85A-sHA2 or pHA were significantly higher than those of their respective controls *(P* < 0.05).

The survival rates were monitored from Day 1 after infection with IAV. The survival rates were high in the pAg85A-sHA2 and pHA groups whereas they were low in the other groups. There were significant differences between the survival rates in the pAg85A-sHA2 group and pHA group compared with the others (*P *< 0.05) (Figure 6). The survival rate of mice vaccinated with pAg85A‑sHA2 was significantly higher than that of mice vaccinated with psHA2 (*P *< 0.05). The survival rate of mice vaccinated with psHA2 was higher than that of mice vaccinated with pAg85A, pEGFP-C2 or PBS. Furthermore, no significant differences were observed between survival rates in the pAg85A-sHA2 group and the pHA group (*P *> 0.05) (Figure 6). 

**Figure 7:**
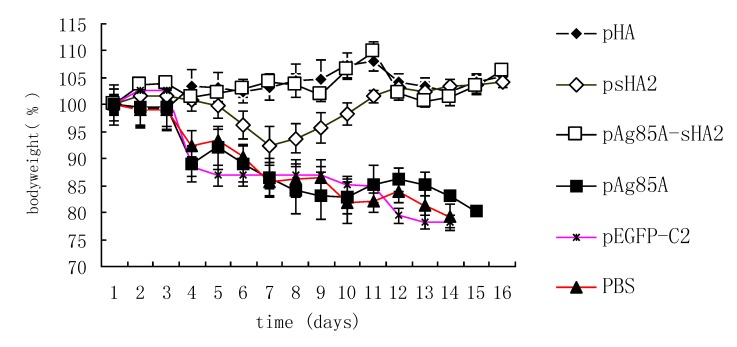
Body weight changes of mice after PR8 virus infection. Weight was calculated relative to that at Day 0. Data are presented as means ± SEM of mice per group. Marked body weight loss was detected 48 h after infection. While body weights significantly decreased in the PBS, psHA2, pAg85A and pEGFP-C2 groups, those of the pAg85A-sHA2 and pHA groups did not change.

Body weight loss could be used to follow the course of IAV infection in mice. Marked body weight loss was detected 48 h after infection. While body weights significantly decreased in the PBS, psHA2, pAg85A and pEGFP/C2 groups, those of the pAg85A-sHA2 and pHA groups did not change, indicating a clinically protective effect by the fusion of the DNA vaccine vector that was comparable to that of the pHA vaccine vector (*P* < 0.05 *vs.* mice of PBS group, psHA2 group, pAg85A group and pEGFP/C2 group) (Figure 7). The body weight loss of mice vaccinated with psHA2 was significantly lower than that of mice vaccinated with pAg85A, pEGFP-C2 or PBS (*P *< 0.05).

**Figure 8:**
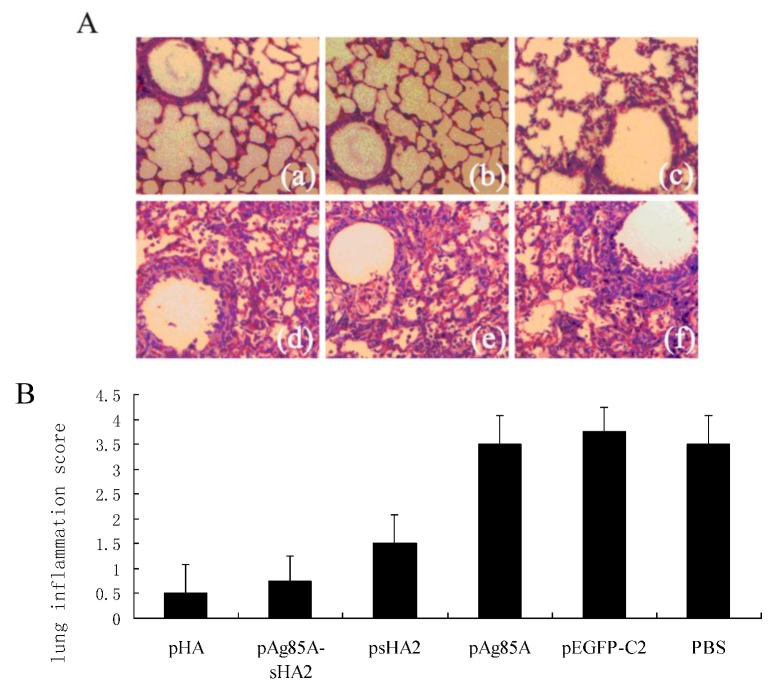
Histopathological analyses of the mouse lungs on the 8th day after the PR8 virus challenge. (**A**) Representative lung slides from all groups (100×). (a): pHA; (b): pAg85A-sHA2; (c): psHA2; (d) pAg85A; (e): pEGFP-C2; (f):PBS. Mice were immunized with pHA, pAg85A-sHA2, psHA2, pAg85A, pEGFP-C2 or PBS. Histopathological analyses of the mouse lungs was performed. The alveolar inflammatory cell numbers were significantly reduced in the psHA2, pAg85A-sHA2 and pHA groups. (**B**) Semiquantitative histology scores of all groups. Lung inflammation was significantly reduced in the psHA2, pAg85A-sHA2 and pHA groups (Figure 8) (*P *< 0.05).

Lungs were harvested on Day 8 after infection with the PR8 virus to prepare hematoxylin and eosin (H&E)-stained lung slides for histopathological examination. The alveolar inflammatory cell numbers were reduced in the psHA2, pAg85A-sHA2 and pHA groups (Figure 8). Lung inflammation was significantly reduced in the psHA2, pAg85A-sHA2 and pHA groups (Figure 8) (*P *< 0.05).

## 3. Discussion

While antibodies against HA are the major determinants of host protection against IAV infection, HA can also induce high frequencies of effector and memory CD4 T cells [1]. The variability of IAV, particularly that of the HA1 subunit, is one of the major difficulties in developing prophylactic vaccines. Therefore, the development of an IAV vaccine that can induce immune responses to conserved domains, such as the HA2 subunit, would be highly advantageous.

In this study, we developed a DNA vaccine vector encoding the Ag85A-sHA2 fusion protein with the aim of enhancing the protection against IAV that is afforded by the HA2 immunogen alone. Following the confirmation of successful expression in mammalian cells, the novel vaccine vector and corresponding controls were used to immunize mice via the intramuscular route before the challenge with the PR8 virus. Interestingly, while the sera from mice vaccinated with the pAg85A-sHA2 fusion vaccine vector exhibited apparently low functional antibody titers in the HI test, they were relatively highly effective in the neutralization assay. Furthermore, the viral loads of the mice vaccinated with the fusion construct (pAg85A-sHA2) were the lowest after challenge compared with those of the other groups given DNA vaccine vectors expressing either Ag85A or HA2 epitopes alone. The HI antibody titers were low in these groups for both the primary and booster responses, as might be expected based upon the absence of the HA1 antigen in the vaccine vector, which could induce antibodies against the receptor binding site or other epitopes involved in HI. This observation is consistent with a previous study describing NAbs to HA2 epitopes that could not completely prevent the hemagglutination of erythrocytes [7]. It is speculated that such antibodies may prevent infection at a step other than that of the initial virus attachment to the target cells [7], although the mechanism has not yet been fully explained. The epitopes of sHA2 exist in many homologous strains, as showed in Table 2. As shown in Figure 3C, the IgG antibodies induced by pAg85A-sHA2 could bind recombinant H5 HA proteins of the A/Anhui/1/2005 better, compared with those induced by pHA. Nevertheless, our results demonstrated that the humoral immune responses to HA2 epitopes were successfully induced by the pAg85A-sHA2 and psHA2 vaccine vectors and the IgG antibody induced by pAg85A-sHA2 or psHA2 could bind recombinant H5 HA proteins of the A/Anhui/1/2005, although further tests are warranted to determine whether these responses to HA2 epitopes may confer protection against diverse types of IAV.

The sHA2 construct did not include a signal peptide. The posttranslational processing of sHA2 did not involve its secretion into the extracellular space. We assumed that the high expression of the sHA2 ORF in our experiments, which was driven by the plasmid’s strong cytomegalovirus (CMV) promoter, led to the release of free sHA2, which was responsible for the induction of antibodies and cell damage.

Our analysis of cytokine production showed that the sera IFN-γ levels in the psHA2 group were significantly higher than those in the pEGFP-C2 and PBS groups after PR8 virus challenge. IFN-γ is a representative Th1 cytokine associated with inhibition of IAV proliferation [13]. The alveolar inflammatory cell numbers were reduced in the psHA2 group. These results confirmed that sHA2 alone could provide limited protection against the PR8 virus. 

Importantly, vaccination with the pAg85A-sHA2 fusion construct produced significantly higher sera IFN-γ levels and resulted in higher survival rates than vaccination with pHA2. The IAV titer from the pAg85A-sHA2 group was lower compared to those from the pEGFP-C2, psHA2 and pAg85A groups. The alveolar inflammatory cell numbers were reduced in the psHA2, pAg85A-sHA2 and pHA groups. The increased survival rate after challenge in the pAg85A-sHA2 group was likely due to Ag85A-mediated immune activation, which enhanced the protective effects of sHA2 against PR8 virus challenge, thereby decreasing the viral load. As IAV can suppress the functions of immune cells [20,21], the elimination of IAV in our experiments may have thus resulted in the enhancement of these functions. Finally, the decreased viral loads relieved pneumonia in mice, as determined by the histopathological analysis. 

In addition to analyzing the IFN-γ responses above, we also investigated the IL-4 levels following vaccination with the various constructs. IL-4 is a cytokine of the Th2 response [22]. Sera IL-4 levels were significantly higher in the pAg85A-sHA2 group or the pHA vaccinated group compared to those in the psHA2 group (*P* < 0.05) on Day 12 after infection with the PR8 virus. These results demonstrated that pHA or pAg85A-sHA2 vaccine could increase the Th2 cytokines response. 

HA2 is masked by the membrane-distal portion of HA, which is a bulky and highly immunogenic globular head domain [8]. The HA2 subunit is relatively conserved [6,7]. Experiments are ongoing to determine whether the responses to HA2 epitopes may confer protection against diverse types of IAV. The preliminary results, which will be reported in a future paper, have shown that the antibodies against sHA2 epitopes can bind H5 HA.

## 4. Materials and Methods

### 4.1. Design of Epitopes

The HA (ABO21709.1) and Ag85A (AY207395.1) sequences were downloaded from the National Center for Biotechnology Information (NCBI). The B-cell and T-cell epitopes for HA2 and Ag85A were predicted bioinformatically using the network servers BCEPRED [23] and SYFPEITHI [24], respectively. With the NCBI Blast algorithm [25], conserved epitopes were clearly identified in HA2 between amino acids (aa) 350 and 498 in the N-terminal region of HA Subtypes 1, 5 and 6 (Table 2). The predicted results were similar to those that were reported by Sui *et al.* [7]. The short HA2 (sHA2) sequence (from aa 350 to 498) was used to construct the novel vaccine vector. The immunodominant epitopes of Ag85A were identified within aa 1 to 250 of the N-terminal sequence (data not shown), which was consistent with previous reports [14,15].

**Table 2:** Predicted B and T cell epitopes in sHA2.

Epitope	Region	Amino acid sequence
B epitope	H1HA_368–375_	YHHQNEQG
B epitope	H1HA_379__–386_	YAADQKSTQ
B epitope	H1HA_391__–397, _H5HA_347__–353_	GITNKVN
B epitope	H1HA_453__–460_, H5HA_455__–462_	DFHDSNVK
B epitope	H1HA_491__–498_, H5HA_493__–500_	ECMESVRN
T epitope	H1HA_373__–387_	EQGSGYAADQKSTQN
T epitope	H1HA_439__–453_	NAELLVLLENERTLD
T epitope	H1HA_434__–448_	DIWTYNAELLVLLEN

### 4.2. Construction of Recombinant Eukaryotic Expression Plasmid Vaccine Vectors

The pEGFP/Ag85A (pAg85A) plasmid was constructed by first cloning the Ag85A sequence (aa 1 to 250 of the N-terminus) [14,15,16] from BCG, and pEGFP/sHA2 (psHA2) was constructed by cloning the sHA2 sequence (aa 350–498 of the HA N-terminus) from the IAV strain A/PR/8/34 (H1N1) as described previously [17]. These sequences contain the immunodominant epitopes of Ag85A and sHA2. The hydrophilicity of the proximal 250 amino acids of Ag85A is similar to that of the HA N‑terminal amino acids 290–321 [16]. Ag85A and sHA2 were fused using a 5 glycine linker as described previously [8] and the resulting fusion was designated as Ag85A-sHA2. The pEGFP/Ag85A-sHA2 (p Ag85A-sHA2) expression vector was constructed by inserting Ag85A-sHA2 into pEGFP-C2 (Clontech, Houston, TX, USA). The pEGFP/HA (pHA) expression vector was constructed by inserting HA (Reference Sequence: NC_002017.1) into pEGFP-C2. The successful insertion of all fragments was confirmed by restriction endonuclease digestion. Sequencing was performed using an ABI Prism 377XL DNA sequencer. Large scale extraction and purification of pEGFP-C2, pHA, pAg85A-sHA2, pAg85A and psHA2 were performed using the EndoFree Plasmid Maxi Kit (Qiagen, Düsseldorf, Germany). 

### 4.3. Cell Transfection Assay

Using the Lipofectamine^TM^ 2000 transfection reagent (Invitrogen, Carlsbad, CA, USA), 0.8 μg of each of the purified eukaryotic expression plasmids pHA, pAg85A-sHA2, pAg85A, pEGFP-C2 and psHA2 were transfected into 1 × 105 HEK293 cells (CRL-1573™) obtained from the American Type Culture Collection (ATCC) according to the manufacturer’s instructions. Each plasmid was transfected in triplicate wells, and PBS was applied as a mock transfection control. Plasmid expression in all sets of transfected cells was assessed by fluorescence microscopy at 48 h after transfection.

Total RNA from each set of transfected cells was isolated by using 1 mL TRIpure Reagent according to the manufacturer’s instructions (Biotake, Beijing, China). Contaminating genomic DNA was then eliminated from the RNA preparations by using RNase-free DNase I (Biotake, Beijing, China). Reverse transcription (RT) was performed with the ReverAid^TM^ H Minus First Strand cDNA Synthesis Kit (Fermentas, Pittsburg, PA, USA). cDNA samples were amplified by PCR using 2× TransTaq High Fidelity (HiFi) PCR SuperMix (Transgene, Beijing, China) in a 50 μL reaction mixture according to the manufacturer’s instructions. PCR primer pairs for mouse GAPDH were obtained from the RevertAid^TM^ H Minus First Strand cDNA Synthesis Kit (400 bp). The following PCR primer pairs were used: pAg85A-sHA2, 5'-CCCTCGAGCATGCAGCTTGTTGACAG-3' and 

5'-CGCGGATCCTCATTTGGGGTAATCATAA-3'; 

pAg85A, 5'-CCCTCGAGCATGCAGCTTGTTGACAG-3' and 

5'-CGGGATCCTTAGCCGTTGCCGCAGTACAC-3'; 

psHA2, 5'-CCGCTCGAGTATTGAAGGGGGATGGA-3' and 

5'-CGCGGATCCTCATTTGGGGTAATCATAA-3'; and 

pHA, 5'-CATAAGCTTAACCAAAATGAAGGCAACC-3' and 

5'-CGCGGATCCTCATATTTCTGAAATTCTAAT-3'. Control PCRs using glyceraldehyde 3-phosphate dehydrogenase (GAPDH) primers were performed on cDNA from the pHA, pAg85A-sHA2, pAg85A, pEGFP-C2 and psHA2 groups. Test PCRs for the pEGFP-C2 group were performed with the pHA primers, psHA2 primers, pAg85A primers and pAg85A-sHA2 primers. Test PCRs for the pHA, pAg85A-sHA2, pAg85A and psHA2 groups were performed with their respective primers. All PCRs were performed simultaneously under the same conditions as follows: five minutes at 95 °C, followed by 30 cycles consisting of 30 s at 95 °C, 30 s at 50 °C and 2 min at 72 °C. Following the amplification, 10 μL of each PCR product were subjected to electrophoresis on a 2% agarose gel and visualized by ethidium bromide staining. PCR amplification and sequencing were performed using an ABI Prism 377XL DNA Sequencer.

### 4.4. DNA Vaccination and Challenge Infection with the PR8 Virus

A total of 120 female BALB/c mice, 4–6 weeks old, were purchased from the animal care center of Sichuan University and divided into six groups, with 20 mice in each group. They were bred under pathogen-free conditions. Each group of mice was injected separately with one of the following: pAg85A-sHA2, pAg85A, psHA2, pEGFP-C2, PBS or pHA. The mice were immunized by intramuscular injection in the quadriceps of both legs (50 μg plasmid in 50 μL PBS per leg) twice with a 3-week interval between injections. The pHA group was the positive control group.

Seven days after the booster immunization, the mice were challenged with IAV, as described previously [17,18]. In brief, the A/Puerto Rico/8/34 virus (H1N1, PR8 virus) was harvested from 10- to 11-day-old embryonated chicken eggs and titrated in a plaque assay. The mice were anesthetized by inhalation of isoflurane and challenged with IAV via intranasal inoculation of ten times of 50% mouse lethal doses (10 × LD50) of the PR8 virus diluted in PBS in a total volume of 50 μL. The survival rates and body weights were monitored daily, and mice losing more than 25% of their initial weights were sacrificed and scored as dead. All of the experiments were performed with institutional approval.

### 4.5. Specimen Collection

Specimens were collected as reported by Zhang *et al.* [17] and Tamura *et al.* [18]. In brief, the first immune serum samples were obtained from each mouse via tail bleeding on day 1 before the booster immunization. The second immune serum samples were obtained from four randomly chosen mice of each group by terminal cardiac bleeding under chloroform anesthesia with chloroform on Day 8 after the virus challenge. Splenocytes from the pAg85A-sHA2, psHA2, pAg85A, pEGFP-C2, PBS and pHA group were isolated and cultured to measure cytokine production. The trachea and lungs were collected and washed twice with 2 mL sterile PBS containing 0.1% BSA. The bronchoalveolar lavages were centrifuged to remove cellular debris, filtered through a 0.22 μM membrane and used for virus titration. Total RNA was isolated from 0.2 mg of left lung tissue from each group using 2 mL TRIpure Reagent according to the manufacturer’s instructions (Biotake, Beijing, China). Contaminating genomic DNA was then eliminated from the RNA preparations using RNase-free DNase I (Biotake). RT reactions were performed with RevertAid^T^^M^ H Minus First Strand cDNA Synthesis Kit (Fermentas, Pittsburg, PA, USA). Lung cDNA samples from all of the groups were stored at -80°C before use. A 0.6 mg left lung samples from each group was used for the histopathological analyses. The third serum samples for the cytokine measurements were obtained from each mouse via tail bleeding on Day 12 after the virus challenge. All of the sera and bronchoalveolar lavages samples were stored at −20 °C before use.

### 4.6. HA Inhibition Assay

The HA inhibition (HI) assay was performed as reported by Zhang *et al.* [17]. In brief, the sera were collected at Day 1 before the booster immunization and at Day 8 after the virus challenge. Two-fold serial dilutions of the sera were prepared in PBS. Four HA units of virus (A/PR/8/34, H1N1) in 25 μL per well were added to a 96-well microtiter plate containing 25 μL of the diluted serum sample, mixed and incubated at 4 °C for 1 h. Chicken erythrocytes in 50 μL (0.5% v/v) were then added to each well and the plate was mixed and incubated at 4 °C for 1 h. The plate was further incubated at 4 °C for 30–45 min. The HI end-point titers were calculated as the reciprocals of the highest serum dilutions that completely inhibited hemagglutination.

### 4.7. Neutralization Assay

The neutralization assay was performed as previously described [8]. In brief, the sera were collected at Day 1 before the booster immunization and at Day 8 after the virus challenge. Fifty microliters of each of the two-fold serial dilutions of treated sera were mixed with 50 μL of the 50% tissue culture-infective dose (TCID50) of the PR8 virus and incubated for 45 min at 37 °C. Madin-Darby canine kidney (MDCK) cells (ATCC, CCL-34) growing in a 24-well plate were washed with PBS and inoculated with the 100-μL virus-antibody mixtures. Following a 45 min incubation, Dulbecco's Modified Eagle's Medium *(*DMEM) supplemented with 2 µg/mL trypsin (Boster Biotechnological, Wuhan, China) and 3% bovine serum albumin (Beijing Biosynthesis Biotechnology Co. LTD, Wuhan, China) were added to each well of the 24-well plate. Each dilution was performed in four replicate wells. The cytopathic effect (CPE) was assayed after incubation at 37 °C for 2 days. The titer from each sample, recorded as the dilution that reduced the CPE by 50%, was calculated by the Reed–Muench method.

### 4.8. Serological Assays by ELISA

Ninety-six-well plates (Costar) were coated with 0.1μg per well of purified recombinant HA protein in PBS. The recombinant HA proteins of the A/Anhui/1/2005 (H5N1, catalog number: 11048-V08H4) and A/PR/8/34 (H1N1, catalog number: 11684-V08H) viruses were obtained from Sino Biological Inc., China. 500-fold dilutions of antisera were incubated on the plates, bound antibody was detected with peroxidase-link Goat Anti-Mouse IgG antibody (Boster Biotechnological, Wuhan, China) according to the manufacturer’s instructions. The experiments were repeated three times, and the results from one experiment are shown.

### 4.9. Cytokine Measurements

Spleens were removed aseptically on Day 8 after the virus challenge and processed as follows. Cells were isolated, washed, adjusted to a concentration of 5 × 106 cells per mL, and grown in a 96-well flat-bottom plate (5 × 105 cells per well) in Roswell Park Memorial Institute medium (RPMI) 1640 medium, supplemented with HEPES (N-2-hydroxyethylpiperazine-N'-2-ethanesulfonic acid), glutamine and 10% heat-inactivated fetal calf serum (FCS). Cells were stimulated separately with 0.5 µg concanavalin A (ConA) or 1.25 µg recombinant HA of A/PR/8/34 (catalog number: 11684-V08H, Sino Biological Inc., Beijing, China) and incubated at 37 °C in a humidified CO2 incubator. After 72 h, supernatants were harvested, pooled and stored frozen at −20 °C until assayed. The experiments were repeated three times, and the results from one experiment were shown. 

Sera were collected on Days 8 and 12 after infection with the PR8 virus. For the cytokine measurements, sera from each group were pooled and stored at −20 °C until assayed. IFN-γ and IL-4 from the sera and IFN-γ from the splenocyte culture supernatants were measured using specific enzyme-linked immunosorbent assays (ELISA; Boster Biotechnological, Wuhan, China) according to the manufacturer’s instructions.

### 4.10. IAV Titrations

IAV titering was performed as reported by Zhang *et al*. [17]. In brief, 10-fold serial dilutions of the sterile bronchoalveolar lavage fluid that were collected on Day 8 after the virus challenge were prepared and used to inoculate confluent monolayers of MDCK cells in 24-well plates. Each dilution was repeated in four wells. The CPE was assayed after incubation at 37 °C for 2 days. The virus titer of each specimen, recorded as the 50% tissue culture infection dose (TCID50), was calculated by the Reed–Muench method. The virus titer from each group was presented as the mean ± SD of all specimens in each group. 

### 4.11. PCR-Amplification and Sequencing of PR8 Nucleic Acids Using RNA Extracted from Lungs of Infected Mice

Lung cDNA samples from each group were amplified by PCR using the 2× TransTaq High Fidelity (HiFi) PCR SuperMix (Transgene, Beijing, China) in a 50 μL reaction mixture according to the manufacturer’s instructions. PCR primer pairs for mouse GAPDH were obtained from the RevertAid^T^^M^ H Minus First Strand cDNA Synthesis Kit. The following IAV NS1 (GenBank: V01104.1) PCR primer pairs were used: 5'- GCTTTCAGGTAGATTGCT -3' and 5'-CCATTCTCATTACTGCTTCT-3'; PCRs for the pHA, pAg85A-sHA2, PBS, pAg85A, pEGFP-C2 and psHA2 groups were performed with GAPDH primers and NS1 primers. All of PCRs were performed simultaneously under the same conditions as follows: Five minutes at 95 °C, followed by the 25 cycles consisting of 30 s at 95 °C, 30 s at 50 °C and 2 min at 72 °C. After amplification, 10 μL of each PCR product were subjected to electrophoresis on a 2% agarose gel and visualized by ethidium bromide staining. PCR amplification and sequencing were performed using an ABI Prism 377XL DNA sequencer.

### 4.12. Histopathological Analysis

Lungs harvested at the 8th day after the virus challenge were used for histological examination. The specimens were fixed in 10% formalin, and embedded in paraffin. Four micrometer sections were stained with H&E. All of the sections were examined for histopathological changes in a blind manner, as described previously [19]. In brief, the entire lung surface was analyzed with respect to the following parameters: pleuritis, bronchitis, edema, interstitial inflammation, percentage of pneumonia and endothelialitis. The pathologist assigned scores to the tissue sections according to these parameters. For lung inflammation and damage, each parameter was graded on a scale of 0 to 4, with 0 meaning “absent” and 4 meaning “severe.” The lung inflammation score for each tissue section was expressed as the average of the scores for all parameters.

### 4.13. Statistical Analysis

Data are presented as means ± SD. Group-group comparisons were analyzed using t-tests. Survival rates were determined using the Kaplan-Meier method. *P* values < 0.05 were considered to be significant. 

## 5. Conclusions

The expression of sHA2 alone could provide a certain level of protection against IAV, but the additional expression of Ag85A from a DNA fusion construct significantly enhanced the protective immune responses to the HA2 epitopes. The pAg85A-sHA2 vaccine vector improved Th1 type responses in the lungs and splenocytes of mice compared with the psHA2 vaccine vector, suggesting that the Ag85A-mediated immune activation was responsible for the increased protection against the PR8 virus challenge. Our findings support the further development of Ag85A as a novel adjuvant for influenza vaccines.
